# Neuroprotective Potential and Underlying Pharmacological Mechanism of Carvacrol for Alzheimer’s and Parkinson’s Diseases

**DOI:** 10.2174/1570159X21666221223120251

**Published:** 2023-05-12

**Authors:** Hayate Javed, Nagoor Meeran Mohamed Fizur, Niraj Kumar Jha, Ghulam Md. Ashraf, Shreesh Ojha

**Affiliations:** 1 Department of Anatomy, College of Medicine and Health Sciences, United Arab Emirates University, PO Box 17666, Al Ain, United Arab Emirates;; 2 Department of Pharmacology and Therapeutics, College of Medicine and Health Sciences, United Arab Emirates University, PO Box 17666, Al Ain, United Arab Emirates;; 3 Department of Biotechnology, School of Engineering & Technology (SET), Sharda University, Greater Noida, UP, 201310, India;; 4 Department of Biotechnology, School of Applied & Life Sciences (SALS), Uttaranchal University, Dehradun, 248007, India;; 5 Department of Biotechnology Engineering and Food Technology, Chandigarh University, Mohali, 140413, India;; 6 Department of Medical Laboratory Sciences, College of Health Sciences, and Sharjah Institute for Medical Research, University of Sharjah, Sharjah, 27272, United Arab Emirates

**Keywords:** Carvacrol, Alzheimer’s disease, oxidative stress, Parkinson’s disease, antioxidant, inflammation

## Abstract

The phytochemicals have antioxidant properties to counter the deleterious effects of oxidative stress in the central nervous system and can be a promising drug candidate for neurodegenerative diseases. Among various phytochemicals, constituents of spice origin have recently received special attention for neurodegenerative diseases owing to their health benefits, therapeutic potential, edible nature, and dietary accessibility and availability. Carvacrol, a phenolic monoterpenoid, has garnered attention in treating and managing various human diseases. It possesses diverse pharmacological effects, including antioxidant, anti-inflammatory, antimicrobial and anticancer. Alzheimer's disease (AD) and Parkinson's disease (PD) are major public health concerns that place a significant financial burden on healthcare systems worldwide. The global burden of these diseases is expected to increase in the next few decades owing to increasing life expectancies. Currently, there is no cure for neurodegenerative diseases, such as AD and PD, and the available drugs only give symptomatic relief. For a long time, oxidative stress has been recognized as a primary contributor to neurodegeneration. Carvacrol enhances memory and cognition by modulating the effects of oxidative stress, inflammation, and Aβ25-35-induced neurotoxicity in AD. Moreover, it also reduces the production of reactive oxygen species and proinflammatory cytokine levels in PD, which further prevents the loss of dopaminergic neurons in the substantia nigra and improves motor functions. This review highlights carvacrol's potential antioxidant and anti-inflammatory properties in managing and treating AD and PD.

## 1 INTRODUCTION

A growing body of evidence shows various health benefits of medicinal plants and their active role in chronic diseases in humans [1]. They are considered safe and cost-effective, and their therapeutic potential has been shown in old cultures and societies of various countries [2]. Medicinal plants such as thyme, olives, turmeric, and oregano have long been employed in culinary preparations and are beneficial in various ailments [2]. Thyme was employed as a preservative, odorant, and flavoring agent in dishes by the Greeks, Romans, and Egyptians. Thyme is a little subshrub widely employed in traditional medicine in the western Mediterranean region; its leaves are used to prepare herbal medicinal products and food additives. The pharmacological properties of thyme include anthelminthic, antioxidative, antifungal, carminative, sedative, antispasmodic, diaphoretic, antibacterial, antifungal, antiseptic, expectorant, and antiviral [2, 3]. Thyme possesses many flavonoids, terpenoids, phenolic acids, and glycosides [4]. Carvacrol (2-methyl-5-(1-methylethyl)-phenol) is an isomer of thymol and a monoterpene phenol, present in various aromatic plants such as *Thymus zygis* (thyme) and *Thymus vulgaris*, *Origanum majorana* (marjoram), *Origanum vulgare* (Greek oregano, wild marjoram), *Origanum dictammus* (dittany of Crete), *Saturejahortensis* (summer savory), *Satureja montana* (winter savory), *Thymbra capitata* (*Spanish origanum*) and *Thymus serpyllum* (white thyme) [5-9]. Carvacrol is broadly used to prepare cosmetic formulations, disinfectants, and fungicide preparations [10]. It is also involved in mosquito control by effectively reducing the hatch rate of their eggs and promoting sterility [11]. Alpha-terpinene and carvacrol have been shown to repel mosquitoes in a human forearm assay compared to the commercial formulation N, N-diethyl-m-methyl benzamide [12]. The chemical structure and physicochemical properties of carvacrol are presented in Fig. (**1**). Carvacrol is a substitute for carbolic acid, creosote, and glycerol of thymol for treating sensitive dentine, odontalgia, and alveolar abscess. In addition, it is also used in the pulp canals of teeth as an antiseptic [13], and in general, it is potentially safe for consumption. The FDA has approved it for use in food, and the council of Europe also added it to the list of chemical flavorings found in baked goods, gelatin pudding, chewing gum, alcoholic beverages, frozen dairy, condiment relish, soft candy, and nonalcoholic beverages [7, 14]. Natural compounds in combination with carvacrol (or carvacrol alone) are effective in minimizing or preventing food spoilage and the growth of harmful microorganisms found in food and are also used as preservatives in food products such as apple juice, grape juice, tomatoes, rice, semi-skimmed milk, honeydew melon, and fresh-cut kiwifruit [5, 14-19]. To inhibit tissue lipid oxidation in poultry meat, carvacrol is added to supplementation feed to improve the nutritional quality of poultry meat [20]. Carvacrol is a potent agonist of transient receptor potential vanilloid 3 (TRPV3) and nonselective calcium-permeable cation channel activated by warm temperature, voltage, and certain chemicals that are robustly expressed in the skin [21], brain, and tongue [22]. Since several studies are involved in developing drugs targeting TRPV, carvacrol may be an agent of pharmacological interest owing to its therapeutic role in developing a pharmacological tool to decipher TRPV3 channel pharmacology. Recently, carvacrol has been an antagonist of TRPM2 and TRPV4 channels, as evident from antagonizing oxidant and apoptotic adverse actions in the neuronal and kidney cells by mitigating oxidative stress (OS)/ADP-ribose (ADPR)-induced TRPM2 and GSK1016790A (GSK)-mediated TRPV4 activations [23]. Carvacrol has been shown to modulate various inflammatory mediators and increase the endogenous antioxidant enzyme level, consequently reducing the deleterious effects of oxidative stress and inflammation-related diseases. These pharmacological properties of carvacrol are required to prevent and treat neurodegenerative diseases, including Alzheimer’s disease (AD) and Parkinson’s disease (PD). This review focuses on oxidative stress and inflammation as the major contributor to the development of AD and PD and the effects of carvacrol in AD and PD by regulating reactive oxygen species (ROS) production and neuroinflammation.

## 2 ROLE OF OXIDATIVE STRESS, INFLAMMATION, AND INFECTION IN THE DEVELOPMENT OF ALZHEIMER’S AND PARKINSON’S DISEASES

Neurodegenerative diseases such as AD and PD represent a major health concern and global economic load on health systems. In general, improved living conditions and health research have increased life expectancy by 30 years in most developed countries [24]. Therefore, the most prevalent neurological diseases in the older population have also increased. Neurodegenerative diseases are late-onset disorders that are prevalent worldwide; by 2050, the total number of AD cases in the world is expected to exceed 100 million. Moreover, the number of cases with PD will also be doubled or even higher than that in countries like China, India, and Indonesia [25]. Due to limited research and knowledge about neurological diseases, the drug development process is very slow; the FDA has not approved any new drug for AD since 2003 [26]. The so-called “anti-Alzheimer’s drug” does not cure it but only delays the progression of the disease [27]. Similarly, there is no robust treatment or cure available for PD. The available drugs or surgery only give symptomatic relief with some side effects. Therefore, urgent research is mandatory to find the potential drug candidate for preventing and treating AD and PD. Neurodegenerative disorders are multifactorial; combining natural compounds or plant extracts as a disease combination therapy can be a promising therapeutic approach. Using phytochemicals or other nature-derived agents as adjuvant or add-on therapy garners attention for combinational therapy for AD and PD. AD is a common progressive neurological illness identified by loss of memory and cognition that eventually leads to impairment in perception, planning, and language. The German psychiatrist Alois Alzheimer first discovered AD in 1906. A sporadic form of AD is more prevalent and is caused by various factors such as head trauma, exposure to environmental toxins, aging, and oxidative stress. Although, the familial form of AD that accounts for 10% of total cases of AD is caused by genetic mutation encoding amyloid precursor protein (APP), presenilin- 1 (PS1), or presenilin- 2 (PS2) [28]. Pathologically, AD is characterized by extracellular deposits of amyloid plaques and intracellular neurofibrillary tangles. Furthermore, tau protein and neurotoxic oligomers of Aβ peptide are the major causes of neurodegeneration. In the familial form of AD, a mutation in three genes, including APP, PS1, and PS2, is primarily involved in AD [29]. PS1 is a member of the γ-secretase complex, which cleaves Notch and APP [30]. The release of varying lengths of the Aβ peptide is caused by presenilin-mediated cleavage of APP. The apolipoprotein (APOE) gene has been discovered as a key genetic risk factor for sporadic AD, with causal mutations in PS1, PS2, and APP [31]. E4 is an APOE isoform, and E4 allele carriers have a greater risk of acquiring AD, with homozygotes developing the disease earlier than heterozygotes [31]. However, the existence of the APOE4 allele has not been proven to be necessary or sufficient for the disease to develop [31]. Mitochondrial failure, inflammation, and oxidative stress are all implicated in the etiology of AD [32-35]. Healthy mitochondria are essential for optimal neuronal function, and oxidative damage in the mitochondria may play a major role in AD development. Oxidative stress is a major cause of neurodegenerative diseases, including AD and PD [36, 37], and is age-related. It is also the initial step in AD development and is also known to play key roles in the neurofibrillary tangles formation [36, 38]. The imbalance between oxidant and antioxidant initiates the events of oxidative stress and the most sensitive organ to oxidative stress is the brain [39] owing to the presence of the high amount of polyunsaturated fatty acid, low amount of antioxidants, and requirement of elevated oxygen supply. Oxidative stress is caused by an imbalance of ROS/RNS (reactive oxygen or nitrogen species), such as OH°, O_2_°^–^ radicals, and nitrogen dioxide radicals (NO°). These free radical species are the product of normal cellular respiration [40, 41], and the accumulation of reactive species in AD causes mitochondrial dysfunctions that disturb the respiratory chain. Therefore, this enhances the excessive production of oxygen free radicals and extracellular Aβ accrual, which initiates the process of inflammation and glial cell activation (another cause of ROS generation). Neuroinflammation plays a key role in the etiology of AD [42]. Glial cells are responsible for the excessive release of cytokines in AD, enhancing the neuroinflammatory processes [43]. Microglia are brain cells that can have beneficial and harmful effects [43]. Microglia are found in an inactive “resting” state in the healthy brain and morphologically identified as ramified cells with tiny somas [44, 45]. Microglia somas are stable in the resting state, though their cellular processes extend and retract to assess their surroundings and make contact with other glial cells and neurons [46-48]. Microglia's protective nature under normal settings is attributed to phagocytosis and neurotrophin release to keep the brain healthy. Microglia becomes activated in response to inflammation or injury/disease and triggers the release of cytokines such as tumor necrosis factor-α (TNF-α), interleukin-1α (IL-1α), and interleukin-1β (IL-1β). Elevated release of ROS and RNS is also caused by microglia under proinflammatory response. At the early stages of AD, proinflammatory mediators increase microglia's activation, eventually leading to synaptic dysfunction and neuronal death [42]. Previous studies reported activation of microglial and elevated release/expression of cytokine in AD [49, 50]. Moreover, the breakdown of the extracellular matrix by the proteolytic enzyme Cathepsin B secreted by active microglia causes neuronal death [51]. Cytokines have been shown to induce the robust synthesis of APP, which further increases Aβ production [49, 52, 53]. Aβ stimulates microglia and the complement system, which produces proinflammatory cytokines and anaphylatoxin, further exacerbating the inflammatory cascade in AD. The activated microglia surround the Aβ plaques and increase the levels of proinflammatory cytokines in the central and peripheral nervous system, implying that inflammation plays a significant role in the development of AD [51]. Recently, quite a few pathogens have been identified as potential causes of AD, but the herpesvirus family has received attention [54]. The herpes simplex virus 1 (HSV-1) is the most widely studied pathogen for AD, owing to the presence of HSV-1 DNA in the brains of patients with AD at autopsy [55, 56]. There are other herpesviruses, such as cytomegalovirus (CMV, Epstein-Barr virus, and human herpesvirus 6 (HHV6), which have also been implicated in AD [57]. The majority of work conducted on HHV6’s showed its ability to seed Aβ plaques *in vivo* and *in vitro* [58]. Readhead and colleagues [59] discovered a large overlap in the affected pathways in HHV6 infection and AD, particularly in APP processing to Aβ peptide, oligomer formation, and eventually amyloid plaque formation. This established a strong mechanistic link between herpesviruses and the development of disease. Furthermore, resident gut microbes may also play a role in the genesis and progression of AD. The gut-brain-microbiota axis has been shown to affect the activity of distant areas, like the brain, through bidirectional contacts of the gastrointestinal tract *via* interactions between the enteric nervous system and the central nervous system (CNS) [60]. Gut bacteria play an important role in the immune system, and dysbiosis may result in inappropriate immunological activation, behavioral difficulties, and a variety of neurological diseases, including AD, which are linked to overactive microglia and increased inflammation. Recently, Minter *et al.* revealed that treating APPSWE/PS1E9 mice with an antibiotic cocktail throughout their post-natal development reduces Aβ plaque deposition and size and gliosis in the region of Aβ plaques [61]. This evidence showed that dysregulation of gut microbiota contributes to systemic inflammation, resulting in amyloidosis and AD progression.

After AD, PD is the second most common progressive neurodegenerative disease. The most common PD symptoms include bradykinesia, rigidity, resting tremors, and mental disorders such as dementia, impaired cognition, and abnormal behavioral changes [62]. Pathologically, PD is identified by the progressive death of dopaminergic neurons and dopaminergic fibers in the substantia nigra and striatum, respectively [63]. In most cases, PD is considered multifactorial, resulting from hereditary and environmental risk factors. Most cases with PD are sporadic, and approximately 10% have a positive family history. Aging is the most significant “environmental” risk factor. Neurodegeneration may be triggered or maintained by age-related changes. Furthermore, a few toxic exposures (*e.g*., MPTP and some pesticides) have been found to cause the death of dopaminergic neurons, and approximately 10-20% of patients have a family history [64]. Recently, multiple variants in an increasing number of genes have been linked to the etiology of PD. Mutations in SNCA (PARK1-4) and LRRK2 (PARK8) are responsible for autosomal-dominant PD forms, while mutations in Parkin (PARK2), PINK1 (PARK6), DJ-1 (PARK7), and ATP13A2 (PARK9) are associated with autosomal recessive forms of PD [65]. Based on their type and position in the mutant protein, mutations in these genes lead to biochemical consequences of loss of function or gain of toxic function. Dopaminergic neurons play key roles in the synthesis and maintenance of dopamine neurotransmitters. Currently, PD medication focuses on maintaining the normal levels of dopamine in the brain. Although this plan to treat and manage PD is useful to some extent, there are some disadvantages to this therapeutic strategy. First, supplementation of higher doses of drugs is required after a while, which causes side effects including dyskinesias, motor fluctuations, and psychosis [66]. In addition, such a treatment causes symptoms of dopa‐resistance in the motor system, such as abnormal posture, gait, and loss of speech, and also in the nonmotor system, such as sleep disorders, insomnia, autonomic dysfunction, and pain and mood impairment over the time [67]. Oxidative stress and inflammation are the primary causes of the development and progression of PD [68]. Elevated oxidative stress has been observed in the substantia nigra of sporadic and familial PD [69]. In normal circumstances, oxidative metabolism produces ROS in the human body. In the brain, some enzymes like tyrosine hydroxylase, L‐amino acid oxidase, and monoamine oxidase (MAO) generate H_2_O_2_ during their enzymatic activity as a typical byproduct. These enzymes play crucial role in dopamine metabolism, which is important in the formation of ROS [70]. However, ROS is produced through various ways in the nigrostriatal dopaminergic system such as mitochondrial dysfunction, dopamine metabolism, inflammation, and reactive iron stored in the neuromelanin, which exacerbate the development of PD. ROS is primarily produced in the brain of patients with PD through mitochondrial impairment in the dopaminergic neurons. Inhibition of the aberrant mitochondrial complex-I lead to the formation of ROS and neuronal death [71]. In an animal model of PD, neurotoxins like 1‐methyl‐4‐phenyl‐1,2,3,4‐tetrahydropyridine (MPTP) [72] and rotenone [73] have been shown to cause oxidative stress and death of dopaminergic neurons *via* inhibition of mitochondrial complex-I. In the familial forms of PD, a mutation in the genes, such as PINK1, α‐syn, DJ‐1, and Parkin, affects mitochondrial functions, integrity, and dynamics. Therefore, changes in the mitochondria lead to the elevated oxidative stress in the dopaminergic neurons [74]. Hence, mitochondrial dysfunction has been linked to the pathophysiology of PD. Dopamine neurotransmitter is stored in the synaptic vesicles and is synthesized in dopaminergic neurons. The outer membrane of mitochondria possesses MAO, an enzyme that catabolizes the excess cytosolic dopamine to ROS [75]. The dopamine levels increase when neurons are damaged or due to levodopa treatment [76]. Another source of ROS production is reactive iron which is stored in neuromelanin. In neuromelanin, iron has low and high-affinity binding sites. Most of this iron binds to high-affinity sites and is stored in an inactive state. When nigral iron levels are increased in the brain of patients with PD, the saturation of high‐affinity sites takes place, and therefore, iron binds to the low‐affinity sites [75]. At low-affinity sites, the iron accumulates in a reactive form and catalyzes the Fenton reaction [77]. The neuromelanin, saturated with iron, also oxidizes dopamine and amplifies the cascade of proteins' oxidative damage [78]. Under normal physiological conditions, neuromelanin is protective, but it becomes deleteriously toxic when excessive amounts of iron accumulate in the substantia nigra. ROS production is also caused by inflammation in the brain. Numerous pathways are causing inflammation in confluence with oxidative stress. In fact, inflammatory responses in microglia are caused by aggregation of proteins and debris of dead neurons, which are the results of oxidative stress. Consequently, microglia produce numerous types of ROS and inflammatory molecules that amplify the cascade of oxidative stress [79]. In PD, inflammation serves as a dual player in molecular events: neuroprotective, neurodegenerative. The ultimate outcome is solely determined by the consistency of proinflammatory and anti-inflammatory reactions. The lethal effects of extended or uncontrolled inflammation on vulnerable neuronal populations can be avoided when trophic factors and anti-inflammatory responses are on the edge. If not, oxidative stress can be induced by inflammatory factors, which allow dopaminergic neurons to induce the death signals [80]. Notably, oxidative stress affects both dopaminergic neurons and microglia. Indeed, activated microglia secretes ROS, RNS, cytokines, and glutamate which make dopaminergic neurons more prone to degeneration [80]. Thus, to attain a further defined and widespread perspective, dopaminergic neurons, and all other cell types, especially microglia, should also be focused on the substantia nigra. Recent investigations have suggested that, in addition to oxidative stress and inflammation, bacterial and viral infections may play a role in etiology of Parkinsonism and idiopathic PD; however, no definitive link has been demonstrated. In contrast to the late onset and progressive development of PD, Parkinsonism caused by the dopaminergic neuronal death due to infectious disease develops quickly [81]. Although infection-related Parkinsonism and idiopathic Parkinsonism are two different diseases, infectious microorganisms have been linked to both the diseases, whether through disease etiology or epidemiological correlations. The Braak hypothesis of sporadic PD states that the disease is caused by an external infection that enters the body through the nose and subsequently travels to the stomach (gut) *via* the vagus nerve, causing alterations in the gut microbiome and the progression of Lewy Body pathology in the gut and the nasal cavity, similar to that observed in viral infection [82-84].

## 3 ANTIOXIDANT AND ANTI-INFLAMMATORY POTENTIAL OF CARVACROL

Essential oils, high in carvacrol, have powerful antioxidant properties [85-88] similar to vitamin E, ascorbic acid, and butyl hydroxyl toluene [89-91]. In the presence of iron (Fe^+3^) and ascorbate, carvacrol reduces phospholipid liposomes peroxidation and superoxide dismutase (a strong peroxyl radicals (CCl3O2) scavenger produced through pulse radiolysis [89]. Low-density lipoprotein (LDL) is inhibited by carvacrol *in vitro* and mediates LDL oxidation within an incubation period of 12 h [89]. Carvacrol effectively scavenges NO from the impulsive breakdown of sodium nitroprusside [92]. Different types of inflammatory processes are mediated by NO. Phytochemicals with phenolic groups have strong antioxidant properties and have been shown to decrease the mortality rate of cardiac disease among people who follow the Mediterranean diet [93]. Furthermore, carvacrol protects against the hepatocarcinogen N-nitroso compound N-nitroso diethylamine by inhibiting lipid peroxidation and improving endogenous antioxidants [94]. Carvacrol plays an important role as an anti-inflammatory agent by suppressing the expression level of cyclooxygenase-2 (COX-2), triggering the peroxisome proliferator-activated receptors (PPAR) α and γ [95], and inhibiting NO production. In the biosynthesis of prostaglandin, COX-2 is a rate-limiting enzyme and has a significant role in inflammation, circulatory homeostasis, and pain. PPARs are nuclear receptor superfamily ligand-dependent transcription factors that control inflammation, energy homeostasis, cell proliferation and differentiation, and lipid and carbohydrate metabolism [96]. PPAR agonist inhibits the mRNA expression for COX-2 and NO synthase, prostanoids, and NO production [97]. Carvacrol inhibits LPS-induced COX-2 mRNA and protein expression in U937 cells and activates PPAR α and γ in bovine aortic endothelial cells [95]. LPS-induced NO production in murine peritoneal macrophages is suppressed by carvacrol owing to its efficiency in inducing PPAR, which eventually leads to decreased NF-κB transcription and iNOS levels [92, 98, 99].

### 3.1 Carvacrol as a Potential Therapeutic Agent for the Prevention and Treatment of Alzheimer's Disease

AD is responsible for 60-70 percent of cases of dementia in the elderly. AD is a neurodegenerative illness caused by the death of neurons in the hippocampus and cortex, resulting in memory and cognitive impairments. Extensive research for medications that help alleviate the disease's symptoms or various slow phytochemicals possesses strong therapeutic potential against neurodegenerative diseases. Among the various monoterpenoids, carvacrol has shown promising pharmacological efficiency against neurological diseases [100, 101]. The essential oil containing 65.27% of carvacrol derived from *Lavandula pubescens* Decne (LP) plants showed strong antioxidant, anticholinesterase, antibacterial, anticandidal, and antidermatophytic activities [102, 103]. Carvacrol derived from LP exhibits strong acetylcholinesterase inhibitory activity (IC_50_ = 1.43 μl/mL) *in vitro*. The common drugs used in AD treatment are based on acetylcholinesterase inhibitors (AChEIs) [104]. The AChEIs play a significant role in memory enhancement in patients with AD by increasing acetyl choline levels in neural clefts and cholinergic transmission in the brain, decreasing the Aβ accumulation and aggregation and eventually preventing the formation of neurotoxic fibrils [105-107]. Carvacrol's acetylcholinesterase inhibitory action is 10 times higher than thymol's, even though the two compounds have a relatively similar structure [108]. Moreover, carvacrol and its several derivatives have shown robust acetylcholinesterase inhibitory effects [109-111]. Carvacrol oil and nanoemulsion of carvacrol significantly ameliorate oxidative stress and inflammation and inhibit the activity of cholinesterase enzyme in AlCl_3-_induced AD in rats [112]; carvacrol nanoemulsion treatment has shown more notable effects compared to carvacrol oil. Furthermore, carvacrol also significantly protects from Aβ_25-35_ induced cytotoxicity in PC12 cells by inhibiting oxidative stress and protein kinase c activity [113]. In addition, carvacrol ameliorates the cognitive impairment caused by intrahippocampal injection of Aβ_25-35_ or intraperitoneal injection of scopolamine in rats [114]. The effectiveness of carvacrol in alleviating the cognitive impairment in these models is because of anticholinesterase, anti-inflammatory, and antioxidant properties. Chronic cerebral hypoperfusion (CCH) is prevalent in various neurological illnesses like AD and vascular dementia [115]. In animal models, carvacrol has significantly improved spatial learning and memory deficits caused by CCH. It also reduces neuronal necrosis and malondialdehyde levels in the hippocampus and increases superoxide dismutase (SOD) and catalase (CAT) activity [116]. The neuroprotective effects of carvacrol against cognitive impairments and its potential in AD are shown in Fig. (**2**).

### 3.2 Carvacrol as a Potential Therapeutic Agent for the Prevention and Treatment of Parkinson’s Disease

Inflammation and oxidative stress are major factors in developing neurodegenerative illnesses, including PD [117-119]. PD is a slowly developing neurodegenerative illness marked by motor and locomotor impairments caused by a disruption in the nigrostriatal dopaminergic system [120]. Carvacrol possesses potent anti-inflammatory and antioxidant properties [85, 89]. Recently, the therapeutic efficacy of carvacrol has been reported in both *in vitro* and *in vivo* models of PD. Carvacrol treatment in 6-hydroxydopamine (6-OHDA) induced Hemi parkinsonian rats have shown improved motor and memory deficit, possibly mediated by its antioxidant potential [121]. In another study, carvacrol protected the 6-OHDA-induced toxicity in PC12 cells in a dose-dependent manner by increasing the cell viability and reducing the intracellular ROS, lipid peroxidation, and a number of annexin-positive cells [122]. Moreover, carvacrol significantly ameliorates bradykinesia, catalepsy, locomotor activity, akinesia, motor coordination, and apomorphine-induced rotations [122]. Another study has shown that carvacrol ameliorates memory loss in 6-OHDA-infused rats but has no effects on contralateral rotation towards lesioned side and hyperalgesia (tail withdrawal latency) [123]. Oral carvacrol supplementation in 6-OHDA injected rats significantly protects against the loss of dopaminergic neurons and also ameliorates the levels of proinflammatory cytokines [124]. The neuroprotective effects of carvacrol against 6-OHDA-induced neurotoxicity are because of its anti-inflammatory and antioxidant effects. In addition, carvacrol reduces the unequal use of forelimbs in mice caused by 6-OHDA [125]. Moreover, it dramatically reduces the loss of tyrosine hydroxylase immunoreactivity in the substantia nigra and striatum. Furthermore, it also reduces the level of caspase-3 and TRPM7 that are increased after 6-OHDA injection in mice [125]. Carvacrol promotes significant neuroprotection in the 6-OHDA model of PD, which may be because of its non-specific blocking impact on TRPM7 channels. The neuroprotective effect of carvacrol has also been investigated in the reserpine-induced PD model [126]. It has significantly protected the reserpine-induced death of dopaminergic neurons and fibers in the substantia nigra and striatum. Moreover, carvacrol prevents catalepsy behavior and the count of vacuous chewing motions, but it does not reverse the reserpine-induced decrease in open-field locomotor activity [126]. Therefore, it may be considered a promising new drug candidate for the prevention and/or treatment of PD. The neuroprotective effects and potential of carvacrol in PD are shown in Fig. (**3**).

## 4 ABSORPTION, DISTRIBUTION, METABOLISM, AND EXCRETION (ADME) AND TOXICOLOGY OF CARVACROL

In rabbits, 1.5 g of orally administered carvacrol is progressively absorbed from the intestines, with approximately 30% of the whole dose remaining in the gastrointestinal system and 25% eliminated *via* urine after 22 h of administration [127]. When carvacrol in sesame oil was given to rats (500 mg) and rabbits (1500 and 5000 mg) *via* gavage, and the amount of carvacrol in the blood, tissues, urine, and feces was tested 2-24 h later, it was found to be distributed in the intestines, stomach, and urine, with tiny levels in the muscle, liver, and the lung. The metabolism of isomeric phenol, carvacrol, and thymol in rats was studied using gas chromatography-mass spectrometric assays and showed rapid metabolite clearance in urine and negligible excretion after 24 h, followed by the absence of metabolites after 48-72 h [128]. Though a sufficient amount of carvacrol and thymol excrete in non-metabolized form, benzyl alcohol, 2-phenylpropanol derivatives, and their corresponding carboxylic acids also form as a result of significant oxidation of the methyl and isopropyl groups. [128]. Studies have shown the bioavailability of carvacrol in the brain tissues as it easily crosses the blood-brain barrier owing to its low molecular weight (150.2 g/mol) and higher lipophilicity [129]. This volatile molecule can accumulate in the brain, interacting with various receptor sites in the central nervous system and exhibiting centrally active properties [130, 131]. Carvacrol has been found to ameliorate behavioral disturbances and DNA damage in the brain of rats exposed to propiconazole [132], inhibit cyclooxygenase enzyme in the hippocampus [133], and oxidative stress in the brain tissues [134]. Recently, numerous formulations have been developed to improve drug formulation with better pharmacokinetic properties. Liposomal formulations, including liposomal suspensions [135], liposomal encapsulation [136], and solid lipid nanoparticles [137], were developed and found bioavailable on oral administration. These formulations exhibit improved solubility, stability, and bioavailability and enhance drug accumulation in the tissues necessary to exert biological effects. The carvacrol-codrugs have been developed by linking the carvacrol hydroxyl group to the carboxyl moiety of sulfur containing amino acids *via* an ester bond [138]. Many novel derivatives of carvacrol have been developed by involving the amide moiety as a linker between the alkyl chains and/or the heterocycle nucleus and demonstrated their acetylcholinesterase and butyrylcholinesterase inhibitor properties [110]. The development, including improved formulations, targeted drug delivery approach, and synthesis of codrugs, encourage future pharmaceutical development and application as nutraceutical or phytopharmaceutical with a pharmacological basis of actions. The toxicological information on carvacrol is limited. Previous studies have demonstrated that carvacrol administered by oral gavage to rats has a median lethal dose of 810 mg/kg of body weight [139]. Carvacrol injected intravenously or intraperitoneally into mice has a median lethal dose of 80.00 mg/kg and 73.30 mg/kg body weight, respectively [10]. No adverse effects have been observed in mice following intraperitoneal injection of carvacrol at the dose of 33.3 mg/kg; however, some nonspecific and slight ataxia were observed at a dose of 50 mg/kg and high doses of carvacrol (110-233.3 mg/kg) caused ataxia, somnolence, and reduced spontaneous motor activity before death [10]. Dermal application of carvacrol to rabbits has shown the LD_50_ at 2700 mg/kg [140]. The LD_50_ of carvacrol after subcutaneous treatment to mice is 680 mg/kg [10], but when it is given to dogs, the lethal dose was found to be 0.31 g/kg [10]. The probable oral lethal dose in humans is 50-500 mg/kg.

## CONCLUSION

Multiple research findings promote the role of terpenoid molecules in improving mental well-being in humans because they are brain-accessible, brain-active, and have a clear influence on neuronal activity *via* neurotransmitter modulation [141, 142], and traditional medicine could be supplemented with these active phytochemicals. The available experimental studies have demonstrated that carvacrol has the potential to be a neuroprotective agent against AD and PD. The data from experimental studies show that carvacrol reduces the manifestations of cognitive impairments, motor dysfunctions, oxidative stress, inflammation, and death of neuronal cells, which is indicative of its multi-targeted preventive potential. Notably, carvacrol has a potent therapeutic impact in lowering the aggregation and accumulation of Aβ, which is one of the classical pathological features of AD. In addition, carvacrol increases the levels of acetylcholine by inhibiting the activity of the cholinesterase enzyme that eventually increases cholinergic transmission in the brain. Most common drugs in the treatment of AD are based on cholinesterase activity. In PD, carvacrol has been proven to prevent the death of dopaminergic neurons, which produces the neurotransmitter dopamine, and is crucial for motor functions. Carvacrol is a multi-targeted molecule compared to other phytochemicals, and a multi-target therapeutic strategy has gradually become a trend in drug development. The available experimental studies demonstrate potential benefits in AD and PD, and no toxicity studies suggest relative safety; the dietary safety of carvacrol-rich plants further supports these findings. Therefore, the available data indicates its use as an adjuvant with currently available modern drugs and may reduce the dose-related adverse effects and maximize therapeutic efficacy. Hence, this rationale can be suggested for pharmaceutical and clinical development. The pathogenic mechanism of AD and PD are complex. Carvacrol can be a powerful pharmaceutical agent for treatment owing to its abundant natural presence, multi-targeted approach, non-toxic agent, and potential to attribute synergistic effects.

## Figures and Tables

**Fig. (1):**
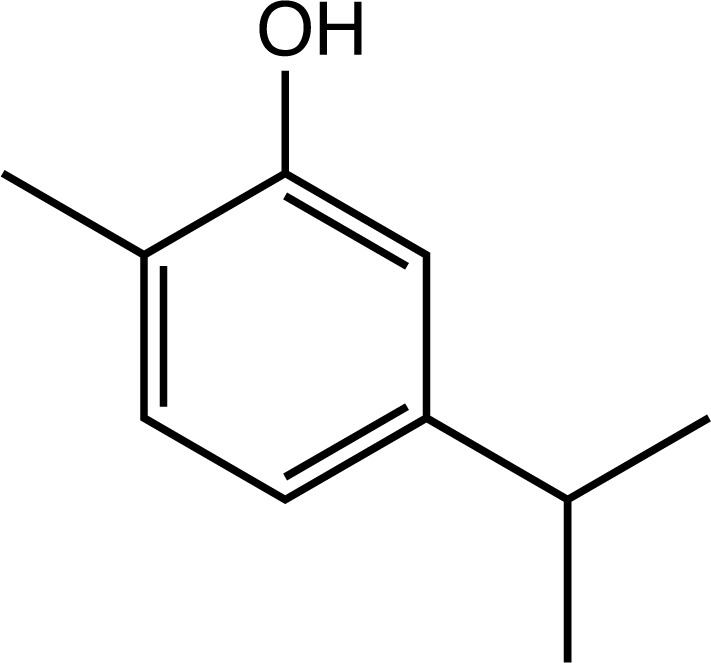
Structure and physicochemical properties of carvacrol: Synonyms: o-Thymol, Antioxine, Isothymol, Oxycymol, Cymophenol, Cymenol, p-cymene-2-ol. Molecular Weight: 150.22 XLogP3: 3.1 Hydrogen Bond Donor Count: 1 Hydrogen Bond Acceptor Count: 1 Rotatable Bond Count: 1 Topological Polar Surface Area: 20.2 Å^2^ Heavy Atom Count: 11 Formal Charge: 0 Physical Nature: colorless to pale yellow liquid, spicy odor Solubility: insoluble in water; miscible in oils and ethanol Density: 0.976 @ 20°C/4°C

**Fig. (2):**
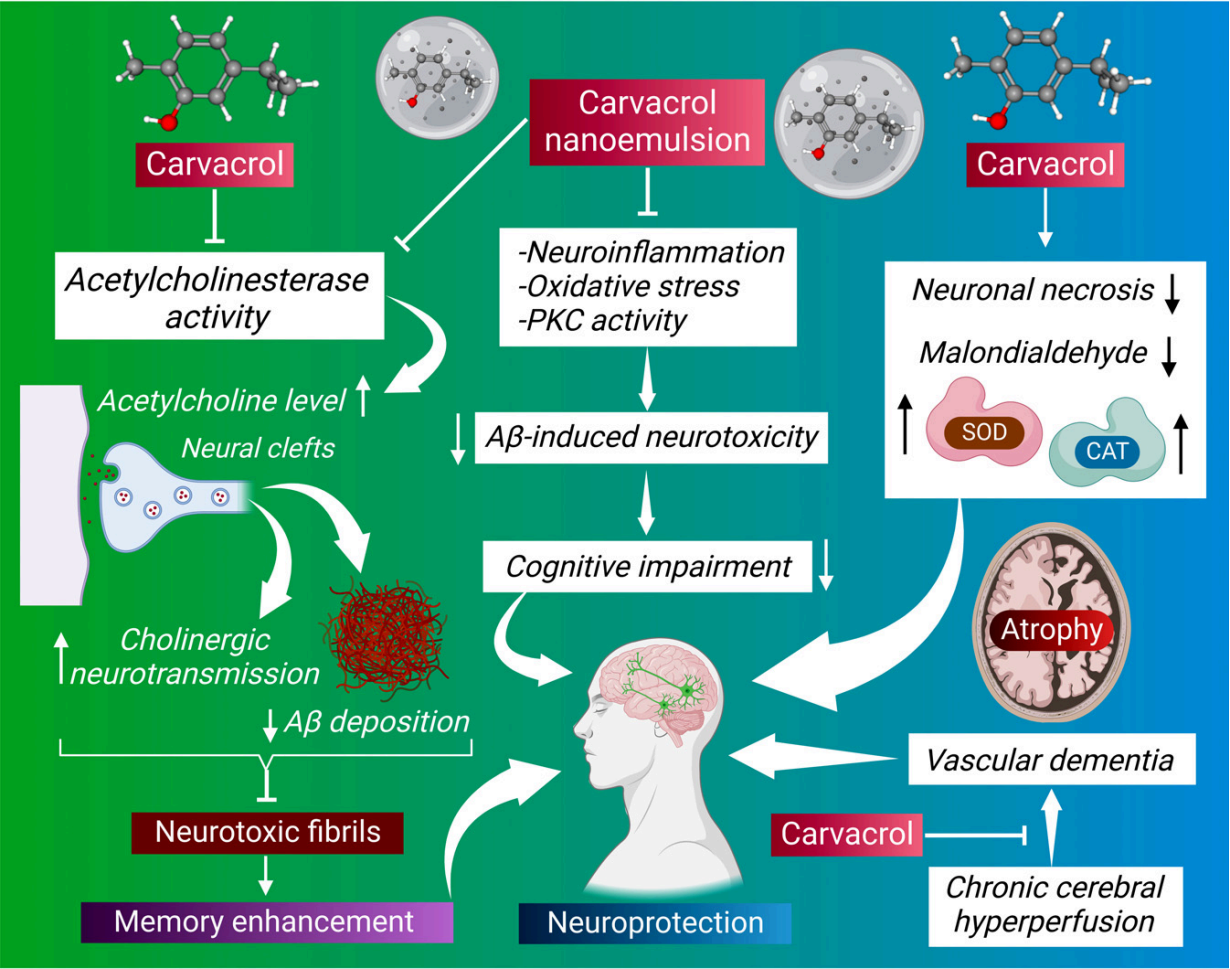
The illustration shows carvacrol's neuroprotective effects against cognitive impairments in AD.

**Fig. (3):**
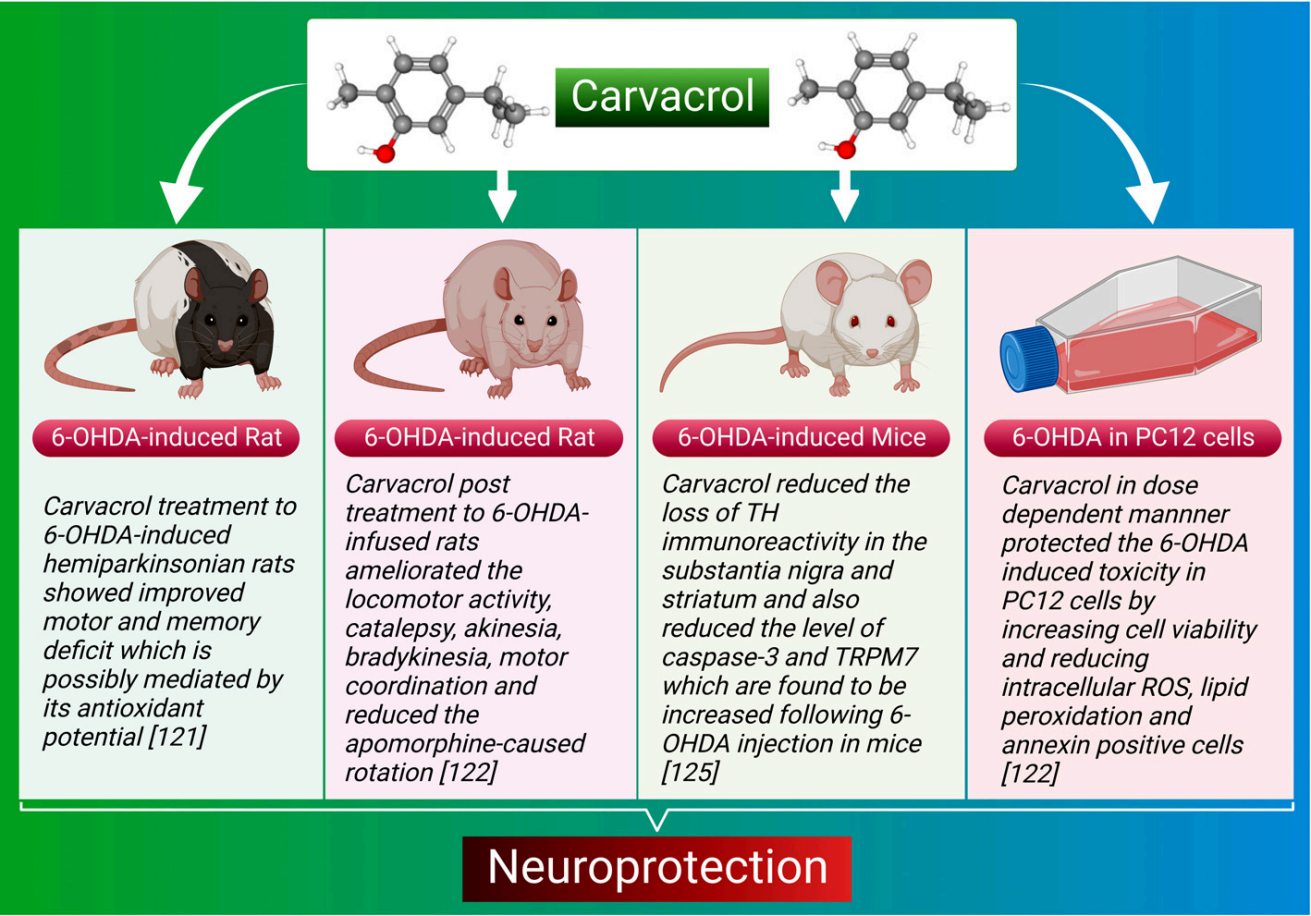
The scheme depicts the neuroprotective effects of carvacrol in PD models.

## LIST OF ABBREVIATIONS

AD: Alzheimer's Disease
APP: Amyloid Precursor Protein
CAT: Catalase
CCH: Chronic Cerebral Hypoperfusion
CNS: Central Nervous System
HSV-1: Herpes Simplex Virus 1
LDL: Low-density Lipoprotein
MAO: Monoamine Oxidase
OS: Oxidative Stress
PD: Parkinson's Disease
ROS: Reactive Oxygen Species
SOD: Superoxide Dismutase
TRPV3: Transient Receptor Potential Vanilloid 3
